# Rearrangement of Actin Microfilaments in the Development of Olfactory Receptor Cells in Fish

**DOI:** 10.1038/s41598-018-22049-7

**Published:** 2018-02-27

**Authors:** Igor V. Klimenkov, Nikolay P. Sudakov, Mikhail V. Pastukhov, Mikhail M. Svinov, Nikolay S. Kositsyn

**Affiliations:** 1 Limnological Institute, Siberian Branch, Russian Academy of Sciences, 3 Ulan-Batorskaya St., Irkutsk, 664033 Russia; 2 Irkutsk State University, 1 Karl Marx St., Irkutsk, 664003 Russia; 3 Irkutsk Scientific Center of Surgery and Traumatology, 1 Bortsov Revolyutsii St., Irkutsk, 664003 Russia; 4 Vinogradov Institute of Geochemistry, Siberian Branch, Russian Academy of Sciences, 1a Favorsky St., Irkutsk, 664033 Russia; 5 Institute of Higher Nervous Activity and Neurophysiology, Russian Academy of Sciences, 5a Butlerova St., Moscow, 117485 Russia

## Abstract

At present, it remains poorly understood how the olfactory neuron migrates through the thick neuroepithelium during its maturation from a stem cell and how it develops a specific sensitivity to environmental odorants after maturation. We investigated the cytochemical features associated with the development of olfactory cells before and after the incorporation of dendrites into the surface of the olfactory epithelium. Using cytochemical staining for the actin cytoskeleton and other cell components, we found that immature neurons acquire a streamlined shape that resembles a «hot-dog» during their migration: a dense layer of actin microfilaments forms beneath the surface membrane of the growing dendrite, and the bulk of the nuclear material moves inside this layer. We have found that when the cell makes contact with its environment, the dendritic terminal develops a wide actin layer, inside which a pore is formed. It is assumed that the functional receptors of odorants generate across this pore the first intracellular signal from environmental water-soluble odorants. These data illustrate the important role of the cytoskeleton in the differentiation of olfactory cells.

## Introduction

Through interactions with environmental odorants, olfaction contributes to the development of complex behaviours in animals and humans throughout their lives. To function reliably, olfaction requires the continuous reproduction of new olfactory sensory neurons (OSNs) in the olfactory epithelium (OE). The OSN pool is replenished through the continuous mitotic activity of globose and horizontal basal cells^1–4^. Despite previous attempts, there is no common understanding of the molecular and cytological bases for the formation of the receptor repertoire acquired by OSNs during their development^5,6^. Understanding the mechanisms underlying the selection of the necessary odorant-binding receptors is particularly important because every organism lives in a specific habitat with a characteristic set of chemical signals, and OSNs should acquire an appropriate level of sensitivity during their development.

Extensive evidence has accumulated showing that cytoskeletal elements play an important role in the adaptive structural rearrangements that occur in various types of cells. The most dynamic cytoskeletal component is the system of actin microfilaments. These filaments have been shown to play a crucial role in cell migration, in the elongation of cell processes, in the transport of organelles and macromolecules, and in the lateral movement and regrouping of adhesion molecules and plasma membrane receptors^7–10^. There are no reports in the literature on the possible involvement of actin in the development, migration, and subsequent differentiation of OSNs. Therefore, the question of which possible actin cytoskeleton rearrangements occur in OSNs in the early and later phases of their development, when their dendrites reach the surface of the epithelium and have the opportunity for primary interaction with the environment, remains unanswered. Addressing this question is important for the identification of mechanisms ensuring the maturation of OSNs when animals are developing adaptive sensitivity to environmental odorants.

This study was conducted on *Cottocomephorus inermis* (Jakowlew, 1890) (Cottidae) (Supplementary Fig. 1), an endemic representative of the ichthyofauna of Lake Baikal. We investigated the cytochemical characteristics of neurogenesis and the participation of actin microfilaments in the migration of young OSNs and their subsequent differentiation after the dendritic terminal incorporates itself into the epithelial surface. As the water of Lake Baikal is very pure, this lake can be used as a unique natural test site at which to collect baseline data, which is a challenge in studies of the sensory systems of aquatic organisms from other, often more polluted, bodies of water. In this study, we demonstrated for the first time, using laser confocal microscopy, that actin microfilaments play key roles in both the early and final stages of OSN differentiation. We found that, to ensure OSN migration, a dense actin layer forms in the near-membrane layer of the dendrite, and the bulk of the nuclear cell material moves into this actin layer. This process endows the cell with a convenient shape for its migration and positioning into the intercellular space of the epithelium.

We also showed for the first time that, when the dendritic terminal makes contact with the epithelial surface, the structural changes in actin microfilaments are accompanied by the formation of a pore. Through this pore, the cell can, for the first time during its development, conduct intracellular signals from odorant receptors (ORs), which bind a limited range of odorants. The results of this study could have important implications for the understanding of the mechanisms underlying the formation of a receptor repertoire in OSNs during their development^11–19^.

## Results

**Figure 1:**
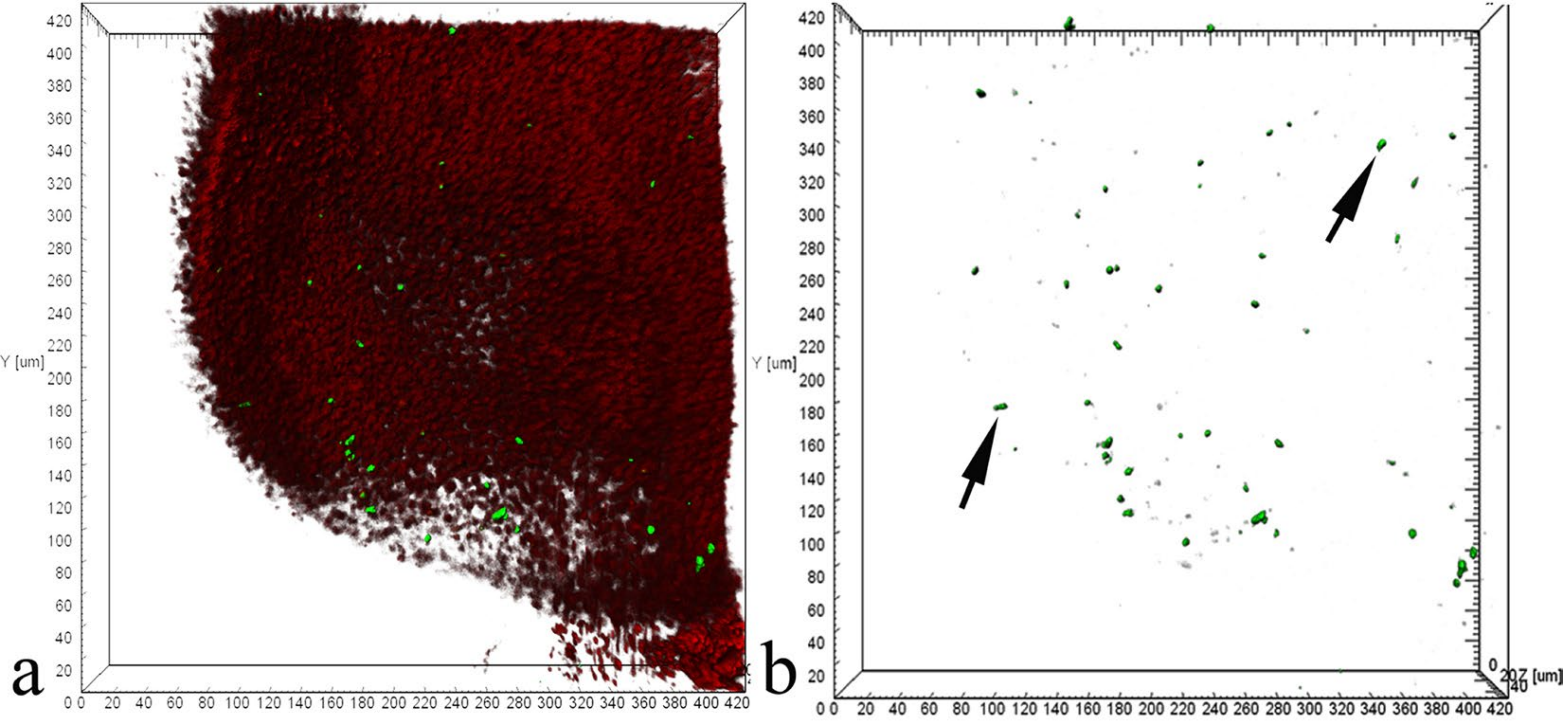
Features of neurogenesis in an individual fold of the olfactory epithelium in *C*. *inermis*. Selective staining of the cell nuclei that incorporated BrdU in the S-phase of the cell cycle (marked by arrows) with FITC-conjugated anti-BrdU antibody (green) 12 h after the BrdU injection. The total cell DNA is stained with 7-aminoactinomycin (red); laser confocal microscopy; 3D reconstructions. (**a**) In the red and green fluorescence channels; (**b**) in the green fluorescence channel.

In *C*. *inermis*, as in other Baikal Cottoidei, the olfactory rosette is 2–2.5 mm in size and has 5–6 folds. The OE has a structure typical of teleosts and consists of receptor, supporting, and basal cells^20–22^. Based on transmission microscopy data, the OE has two types of receptor cells – ciliated and microvillar – of which the former prevail in number^23^. Confocal microscopy shows that within 12 h after BrdU injection, proliferatively active cells are detected in the basal area of all the folds of the olfactory rosette (Fig. 1). A total of 5.06 ± 1.67 cells per 10^5^ μm^3^ incorporated the label. This value corresponds to an average level of proliferative activity in the OE of control teleosts^24,25^. An analysis of preparations stained with FITC-phalloidin for F-actin shows that the daughter cells arising from the division of basal progenitor cells acquire the morphological features of OSNs as they migrate to the epithelial surface.

**Figure 2:**
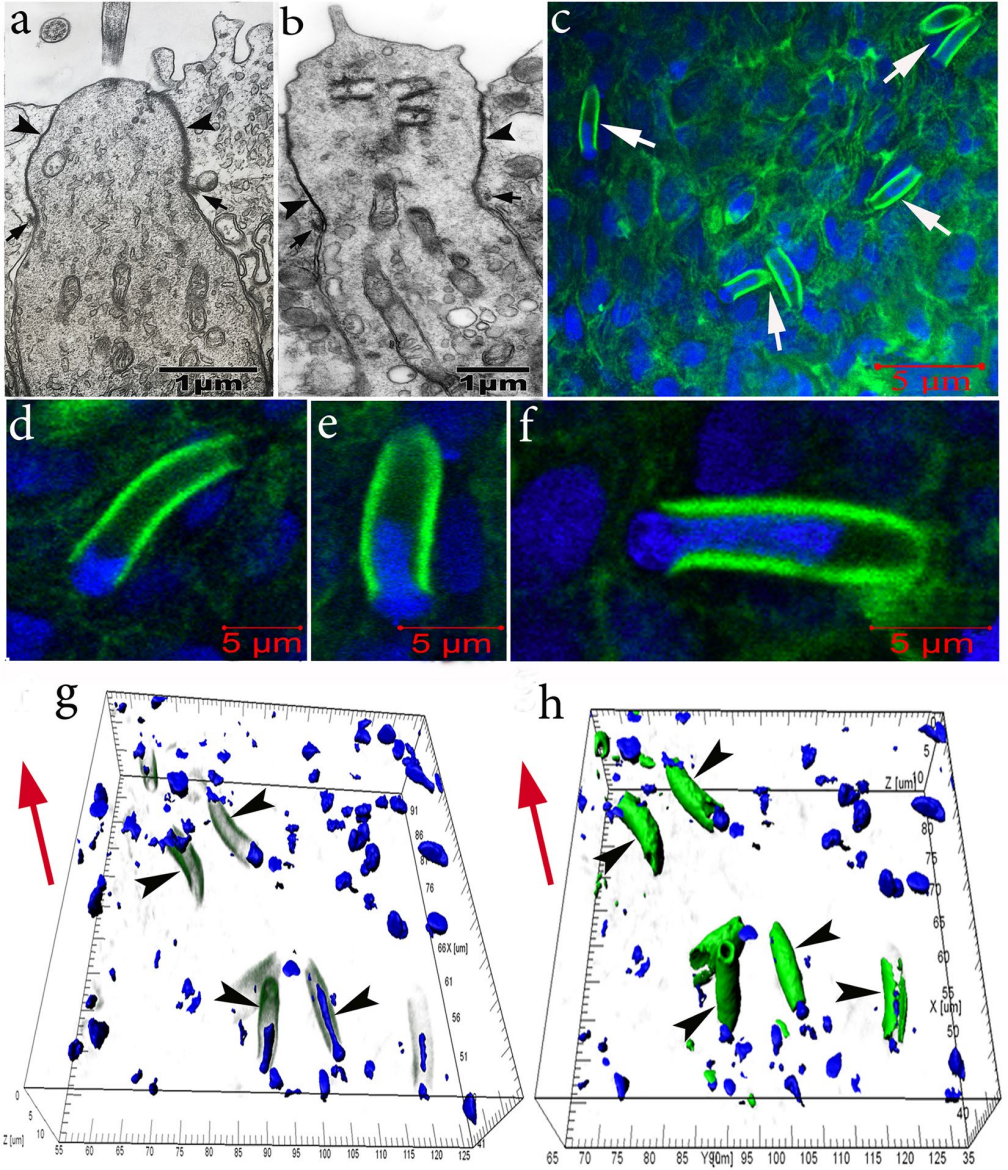
Olfactory sensory neurons in the olfactory epithelium in *C*. *inermis*. Staining for F-actin (FITC phalloidin, green) and for nuclei (DAPI, blue); transmission (**a**,**b**) and laser confocal microscopy (**c**-**h**). (**a**) ciliated OSN; (**b**) microvillous OSN; Big arrows – tight junctions; small arrows – adherens junctions; (**c**) OSN dendrites (marked by arrows) in the epithelium with a high content of near-membrane actin; 2D sections; (**d**–**f**) individual cells with nuclear material, which are immersed, to different degrees, in the inner space of the dendrites (2D sections); (**g**,**h**) several OSNs (marked by arrows) with nuclear material stored inside the actin case with different degrees of fluorescence filtration in the green spectrum (3D reconstructions). The red arrow indicates the direction to the surface of the olfactory epithelium.

In these daughter cells, actin microfilaments are clearly detected in near-membrane regions along elongated processes directed towards the epithelial surface. The width of the near-membrane F-actin layer is 0.45 ± 0.12 μm. Therefore, these cells acquire contours typical of the dendrites of ciliated and microvillar OSNs, which we observed in the OE of *C*. *inermis* by transmission microscopy^23^. The top of a ciliary OSN contains cilia (Fig. 2a), and microvillar cells are equipped with fingerlike growths (Fig. 2b). Figure 2с shows a large group of cells with these dendrites. The diameter of these processes is 3.04 ± 0.49 μm, and their length varies, depending on their development, from 3 to 15 μm, averaging 9.23 ± 4.34 μm. As the dendrites have not yet come into contact with the OE surface, we have identified them as immature OSNs (iOSNs).

**Figure 3:**
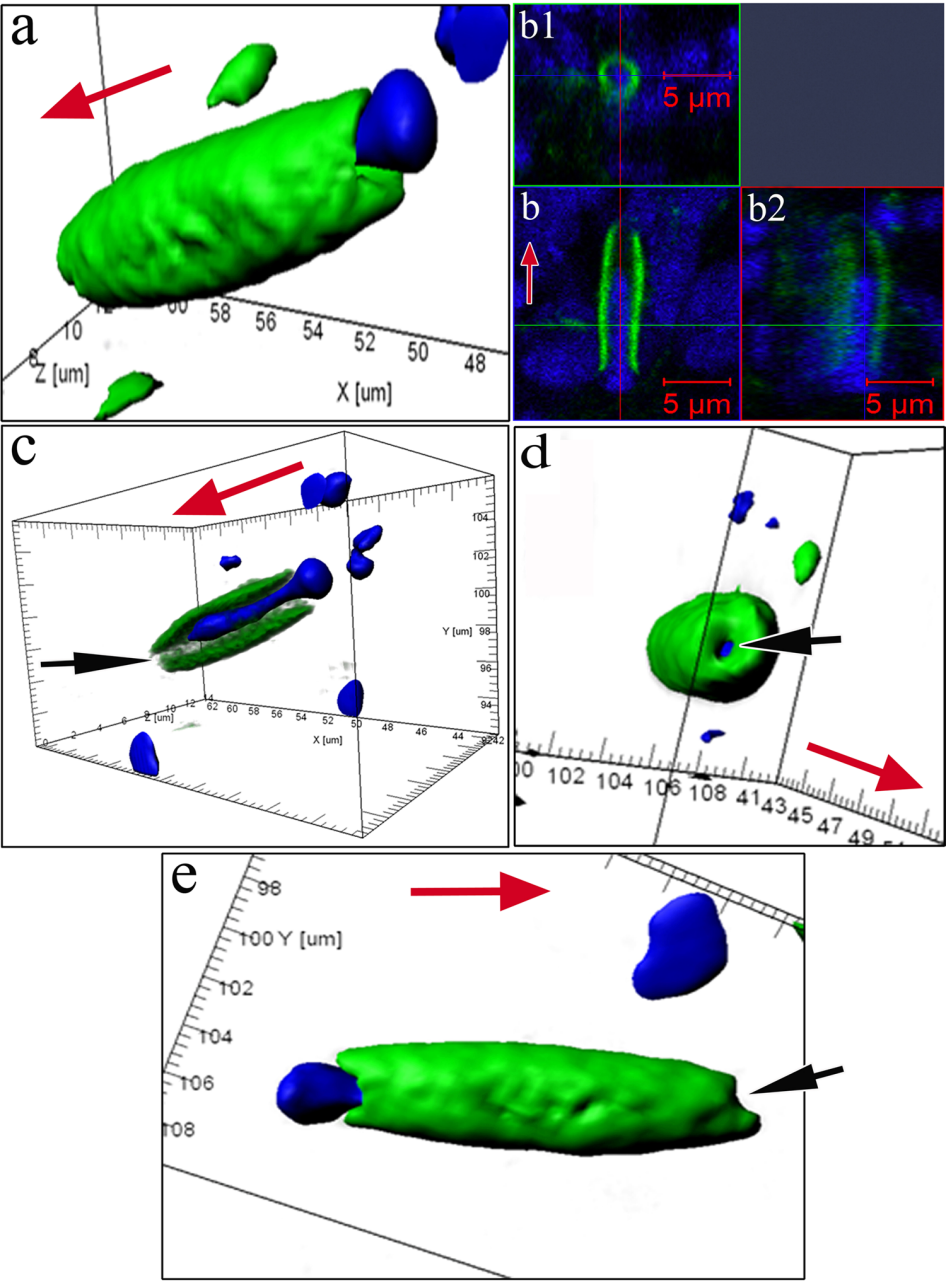
Specific structural organization of OSNs during their differentiation and movement in the olfactory epithelium in *C*. *inermis*. Staining for F-actin (FITC phalloidin, green) and for nuclei (DAPI, blue); laser confocal microscopy. (A) OSN during migration (3D reconstruction); (**b**) A dendrite with nuclear material inside (2D section) and its orthogonal projections (b1 and b2); (**c**) a lengthwise cut through a iOSN; nuclear material is located inside the actin microfilaments; the front section of the dendrite is marked by an arrow; (**d**) apical surface of the iOSN dendrite that contains a section without actin microfilaments (marked by an arrow); (**e**) uneven contours of the front section of the dendrite (marked by an arrow), where the growth cone is presumably located. The red arrow indicates the direction to the surface of the olfactory epithelium.

The formation of a near-membrane actin cytoskeleton is not the only strategy used by iOSNs to migrate from the basal membrane to the upper epithelial layers. The migration of iOSNs between the cells they encounter along the way is accompanied by a reorganization of the shape of the nucleus. A spatial reconstruction of optical sections of dendrites shows that, during cell transport, the nucleus, which is the largest (by volume) organelle in the neuron, acquires an elongated shape because the bulk of the nucleus moves inside the actin casing. In the OE, there exist iOSNs whose nuclei only come into contact with the lower part of the dendrite and still retain their characteristic round shape (Fig. 2d). In other cases, one of the nuclear poles gradually extends to penetrate deep inside the inner space of the dendrite (Fig. 2e,f). Thus, 70–80% of the nucleus is localized inside the dendrite’s actin casing; this redistribution of nuclear material causes the body of the iOSN to resemble a “hot dog” (Fig. 2g,h**;** Supplementary video 1). Only a small portion of the nucleus remains outside the dendrite; the diameter of this portion is comparable to the external diameter of the actin cylinder (Fig. 3а). 2D pictures (Fig. 3b) and orthogonal projections of iOSNs (Fig. 3b1,b2) confirm the assumption that the bulk of nuclear material is localized inside the dendrite. This redistribution likely endows the cell with a more convenient shape for its successful transport within the epithelium. Notably, at this stage of differentiation, the dendrite growth cone contains no actin microfilaments, perhaps because vesicles are delivering and incorporating new membrane material into this region (Fig. 3c–e). Figure 3d shows the apical surface of the iOSN dendrite that contains a section without actin microfilaments (marked by an arrow). The diameter of the pore-like structure is 0.90 ± 0.12 μm.

**Figure 4:**
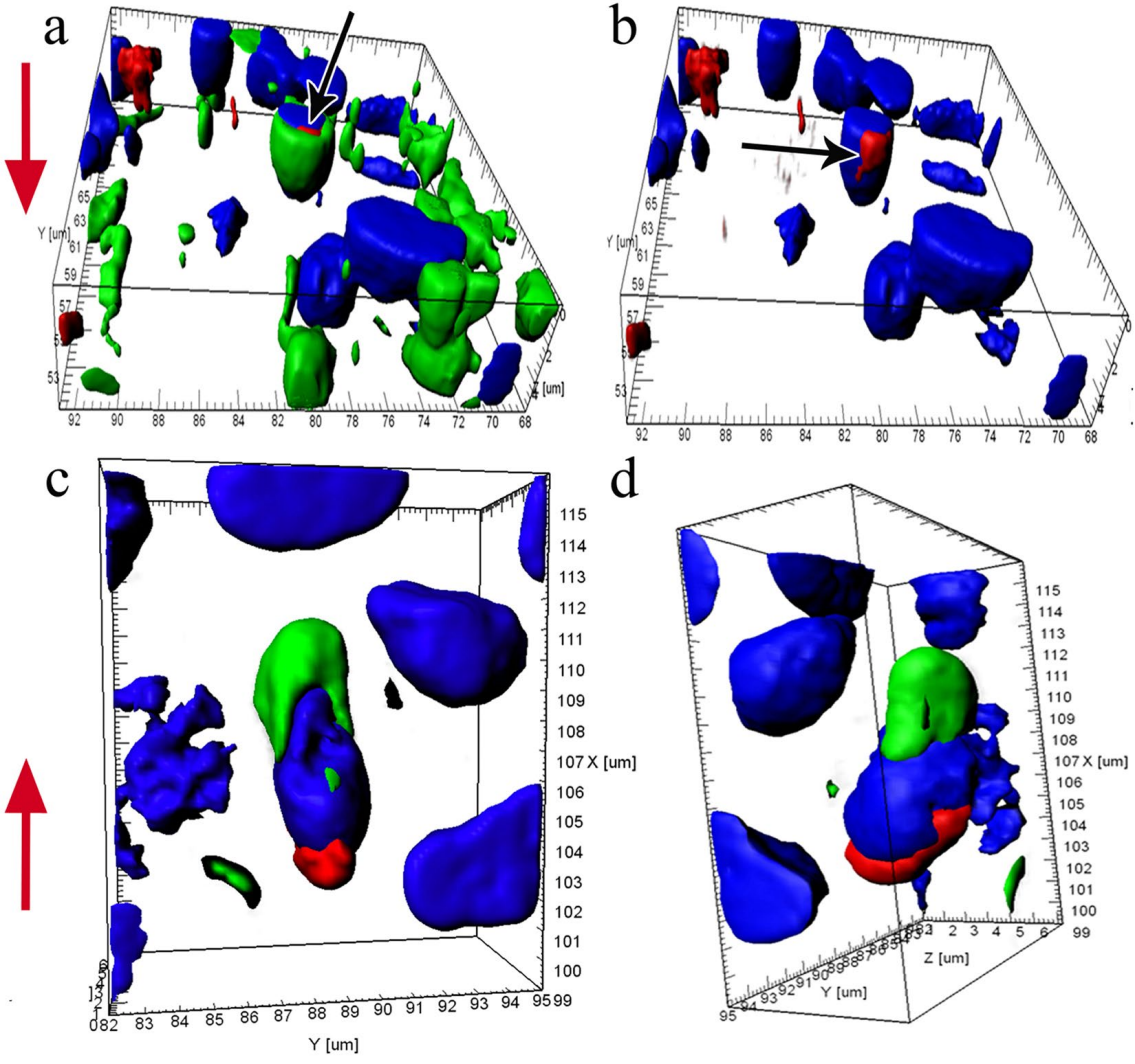
Circumnuclear zones of iOSNs with growing dendrites enriched with multiple actin microfilaments in the olfactory epithelium in *C*. *inermis*; staining for F-actin (FITC phalloidin, green), for nuclei (DAPI, blue), and for mitochondria (MitoTracker®Orange CMTMRos, red); laser confocal microscopy; 3D reconstructions. (**a**) Mitochondria (marked by an arrow) adjacent to the nucleus in the inner space of the dendrite; (**b**) photo in Fig. 4a of the blue and red fluorescence channels (mitochondria marked by an arrow); (**c** and **d**) the cell body with a growing dendrite, at different angles; the upper part of the nucleus is immersed inside the dendrite; the mitochondria are located at the lower pole of the nucleus. The red arrow indicates the direction to the surface of the olfactory epithelium.

In addition to F-actin, iOSNs have functionally active mitochondria inside their dendrites and in the lower poles of their nuclei, which suggests a high level of energy metabolism necessary for cell growth and activity (Fig. 4).

**Figure 5:**
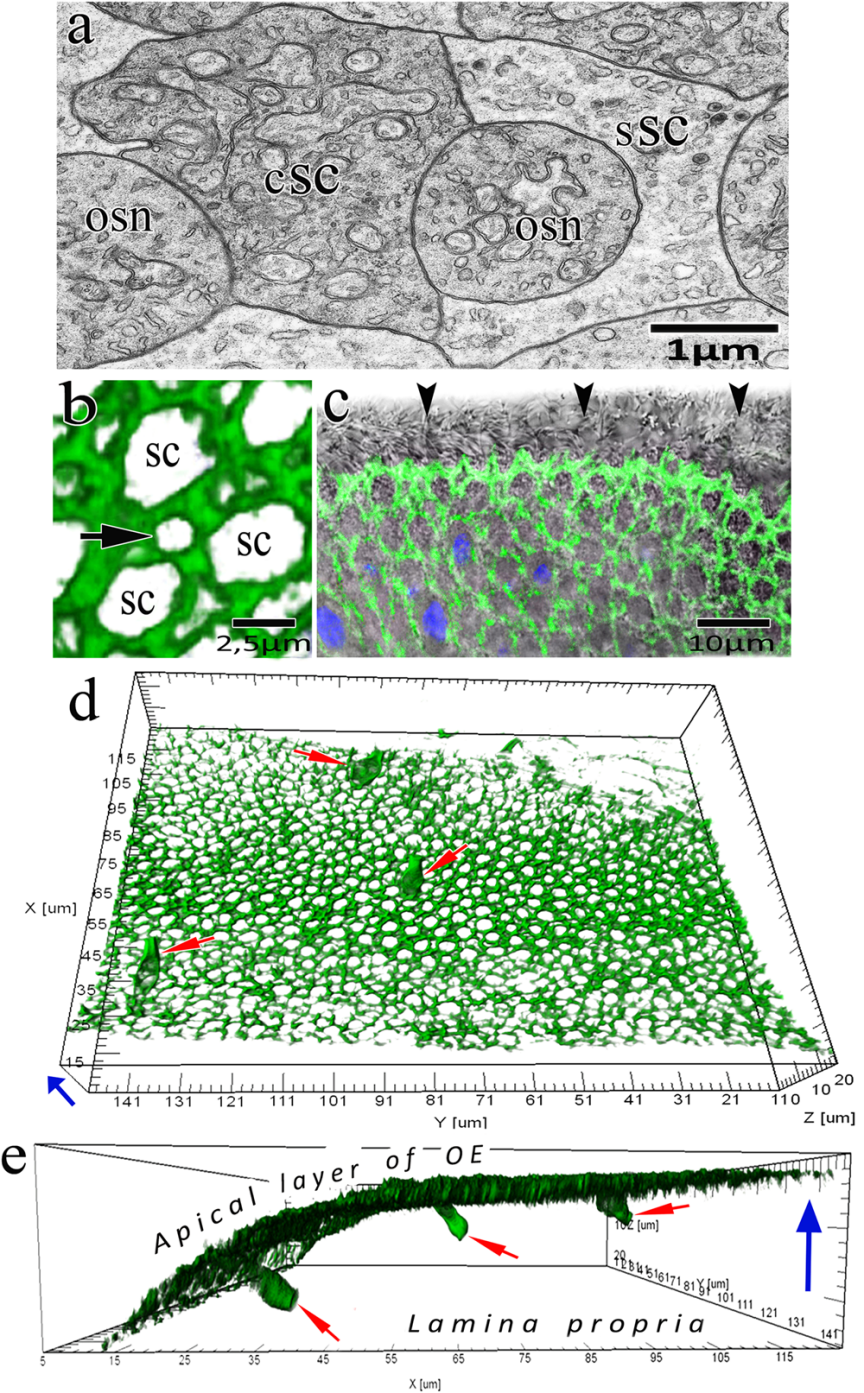
The involvement of actin microfilaments in the structural organization of the olfactory epithelium and the development of receptor cells in *C*. *inermis*. (**a**) Transverse section through OSNs and SCs (transmission electron microscopy); (**b**–**e**) confocal microscopy; staining for F-actin (FITC phalloidin, green); (**b**) Mosaic organization of actin microfilaments in the olfactory epithelium (3D reconstruction); profile of the apical section of a mature OSN marked by an arrow; (**c**) transmitted light mode (the ciliary layer is indicated with arrows); (**d**) Top view of the olfactory epithelium; three OSNs (marked by arrows) incorporated in an ordered fine-mesh network of actin microfilaments in the upper layer of the olfactory epithelium (3D reconstruction); (**e**) the profile in Fig. 5a with the expressed actin cases of OSN dendrites (3D reconstruction); OSNs marked by arrows. Notation: OSN – olfactory sensor neuron; сSC – ciliated supporting cell; sSC – secretory supporting cell. The blue arrow shows the direction to the surface of the olfactory epithelium.

During growth, iOSN dendrites reach the epithelial surface and form tight and adherens junctions (TJs and AJs) with the distal sections of neighbouring cells (SCs or OSNs), thereby integrating into an ordered network of cellular epithelial elements. Moreover, immunocytochemical and immunofluorescent staining of preparations for specific TJ- and AJ-associated proteins clearly reveal the finer rounded contours of dendrites against the background of the surrounding wide supporting cells^26,27^. Studies conducted on different types of tissues show that the actin cytoskeleton is also structurally related to TJs and AJs^28^. In Fig. 2a,b, TJs and AJs are marked by arrows between OSNs and SCs. Staining of the olfactory rosette with phalloidin-FITC allowed the detection of a highly ordered cellular structure of adhesive cell belts formed by actin microfilaments. This inlaid structure is predetermined by the fact that fine OSN peaks are usually intertwined with wider SC profiles in the olfactory epithelium. This structure could be seen clearly in transmission microscopy (Figs. 5a), 3-D reconstructed confocal microscopy (Fig. 5b), and optical fluorescence microscopy (Fig. 5c).

The observed actin-belt network consists of two types of regularly arranged meshes of different diameters: small meshes (1.35 ± 0.14 µm) related to receptor cells and large meshes (3.96 ± 0.38 µm) related to the apexes of supporting cells. We see that, during the growth of the dendritic terminal, iOSNs embed themselves into small meshes while interacting with the apexes of supporting cells (Fig. 5d,e).

**Figure 6:**
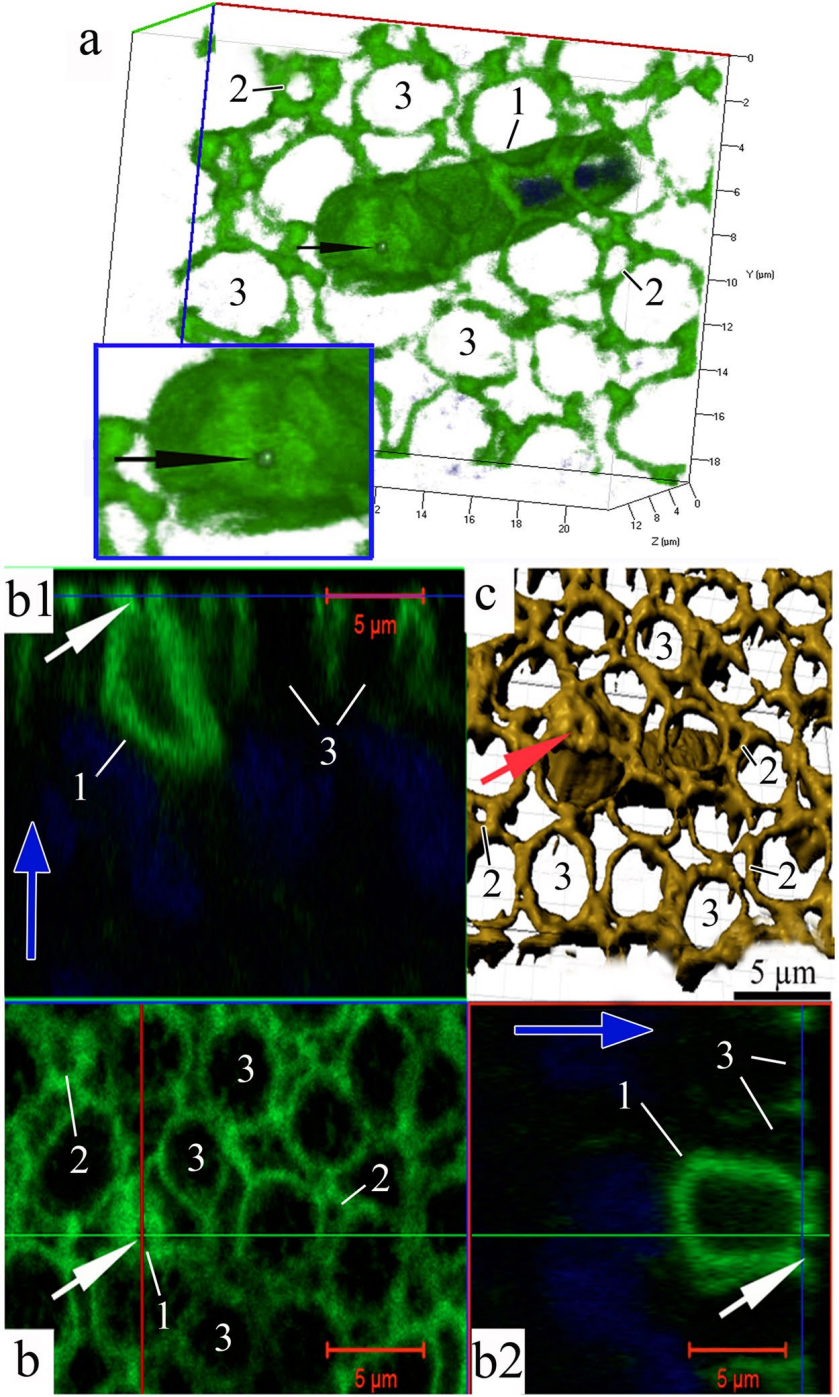
The pore-containing OSN in a wide layer of actin microfilaments in the olfactory knob after the neuron has attached itself to the OE surface, in *C*. *inermis*. Staining for F-actin (FITC phalloidin, green) and for nuclei (DAPI, blue); laser confocal microscopy. (**a**) The OSN is attached to the OE surface between supporting cells; the insert shows an enlarged fragment of the actin case of the dendritic terminal with a pore (marked by an arrow); (3D reconstruction); (**b**) the surface and orthogonal projections (b1 and b2) of the OSN with a pore; (**c**) a 3D reconstruction (surface mode) of the OSN with a pore. Notation: 1 – Dendrites of OSN that contact the surface of the olfactory epithelium; 2 – Profile of the apical section of a mature OSN; 3 – Profile of the apical section of the SC. The blue arrows show the direction to the surface of the olfactory epithelium.

Beginning when the dendrite makes contact with the environment, its apical portion undergoes a series of crucial structural rearrangements. In this developmental phase, an expressed layer of actin microfilaments also forms in the near-membrane layer of the olfactory knob and along the dendrite. This wide (0.45 ± 0.13 μm) continuous layer covers the entire space under the surface membrane of the olfactory knob, except for a small (0.37 ± 0.04 μm) round region containing no actin (Fig. 6; Supplementary videos 2, 3). Calculations show that the area of this actin-lacking region is 2.5 ± 0.5% of the total surface area of the knob. In each petal of the olfactory rosette, we observed 3–6 young OSNs, which were in contact with the epithelium surface and had pronounced actin microfilaments in the layer adjacent to the membrane. Nevertheless, in each petal, there were only 2–3 OSNs in whose top sections we could observe a dense actin microfilament layer with a pore. We were unable to trace the path by which dendrites contacted the surface of the olfactory epithelium and how the transformation of the cytoskeleton led to the formation of a “pore”. We scrutinized orthogonal projections of this specific region, but against the background of surrounding actin microfilaments, this region of the knob produces no fluorescent signal. In a longitudinal optical section, the transparent channel of this pore passes through the entire thickness of the near-membrane F-actin layer and opens into the cytoplasm. Previously, using the “patch clamp” method on isolated OSNs, it has been observed that neurons can react to hydrophobic molecules before their dendrites contact the surface of the olfactory epithelium^29^. Taking this observation into account, we suggest that through the pore-like structure emerging in the apical area of the dendrite after its contact with the environment, the cell acquires the ability to interact with water-soluble odorants for the first time.

**Figure 7:**
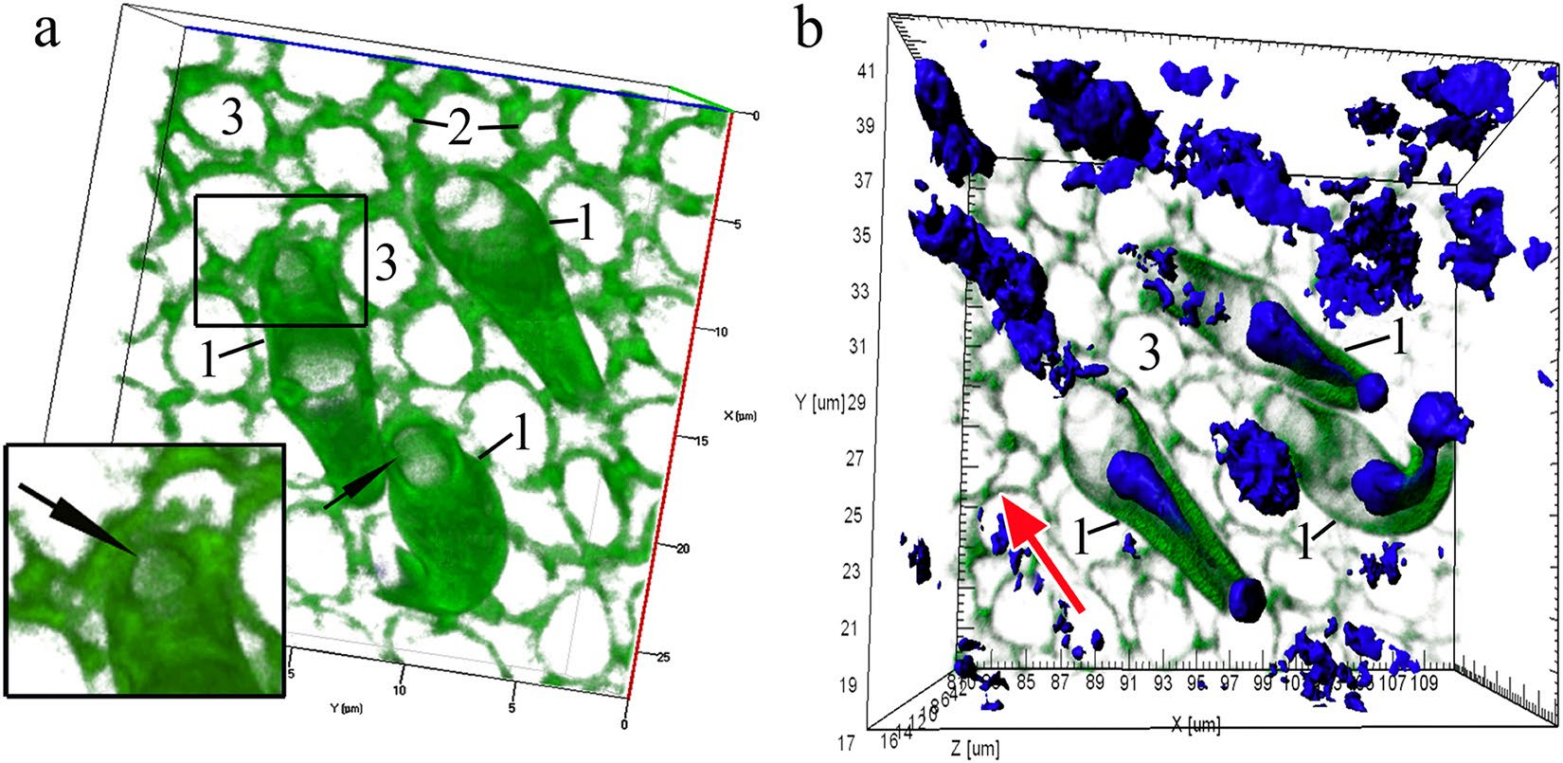
Disappearance of actin microfilaments at the tips (marked by arrows) of three OSNs whose dendrites are attached to the OE surface, in *C*. *inermis*. Staining for F-actin (FITC phalloidin, green) and for nuclei (DAPI, blue); laser confocal microscopy; 3D reconstructions. (**a**) А view of the OSNs from the outer side of the epithelium; the actin-lacking tip of an individual cell is marked by an arrow; the large meshes of F-actin associated with the supporting cells are clear; actin microfilaments in the axial cylinders of the dendrites are partially preserved; elongated nuclear material, the bulk of which is inside the dendrites, is visible; (**b**) a view of the OSNs from the lamina propria of the olfactory mucosa. Notation: 1 – OSN dendrites that contact the surface of the olfactory epithelium; 2 – Profile of the apical section of a mature OSN; 3 – Profile of the apical section of the SC. The red arrow indicates the direction to the surface of the olfactory epithelium.

**Figure 8:**
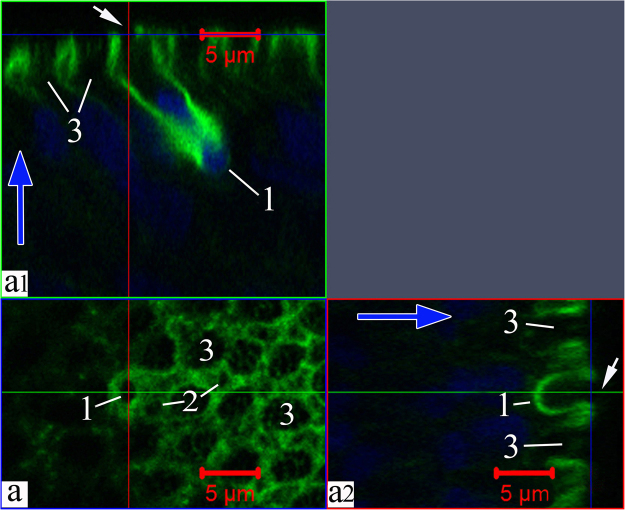
Destructive changes in actin microfilaments in the near-membrane layer of the olfactory knob in an OSN; this layer is partially preserved in the axial cylinder of the dendrite. The bulk of the nuclear material is inside the dendrite. Staining for F-actin (FITC phalloidin, green) and for nuclei (DAPI, blue); laser confocal microscopy (2D section). (**a**) A view of the surface of the olfactory epithelium; a mesh network of actin microfilaments associated with tight junctions is clear; (а1 and а2) orthogonal projections of OSNs. The large meshes formed by actin microfilaments are attributed to the apexes of supporting cells. Notation: 1 – Dendrite of OSN that contact the surface of the olfactory epithelium; 2 – Profile of the apical section of a mature OSN; 3 – Profile of the apical section of the SC. The blue arrows show the direction to the surface of the olfactory epithelium.

During further differentiation, the dense layer of actin filaments near the membrane in the dendrite and its tip gradually disintegrates. Figure 7 (Supplementary video 4) shows three cells with substantially increased pore sizes (up to 2 µm) due to cytoskeletal depolymerisation, with destruction of actin networks also observed in certain parts of the cell body. During further development, the entire wide F-actin layer under the surface membrane of the cell apex is gradually disassembled. This disassembly begins with the olfactory knob and extends over the cell body. This is also evidenced by the patterns of the orthogonal projections of young cells, which show that actin is still present in the near-membrane layer of the cell body, but the dendritic terminal is completely free from actin (Fig. 8). Thus, during differentiation in the OSN, actin microfilaments become clearly structured both during their migration in the epithelium and in the final developmental stages after making contact with the environment.

## Discussion

The received results may be important for the comparison of the cytochemical restructuring process in developing OSNs with receptor function maturation. To study the OSN maturation process, is explored the expressions of different regulatory factors in different periods of OSN morpho-functional development. Earlier studies demonstrated that during mouse embryogenesis, the calcium-binding protein calretinin is expressed transiently in intermediate cells immediately before OSNs become mature and can therefore be used as an indicator of iOSNs^30^.

It has been determined that in mouse cells, single early immature neurons express multiple odorant receptor genes (*Olfrs*), whereas mature OSNs show a high level of expression primarily of single *Olfrs*. Moreover, the expression of the growth-associated phosphoprotein GAP-43 has been distinct only in early immature neurons^17^. Aside from the indicators of iOSNs described above, it has also been recently demonstrated that the mammalian ionotropic serotonin receptor 5-HT_3a_ can be used as a new molecular marker of a proliferative or developmental state, regardless of age^31^. Unlike the results of existing studies on mice, so far, only GAP-43 has been identified as an iOSN histochemical antigen for fish^32,33^. It turns out that calretinin, which is typically present in iOSNs of mice^30^, colours only mature OSNs in fish and may be observed at varying extents in ciliated, microvillar and crypt OSNs^34–40^.

Our studies show that in addition to the previously used differentiation markers, elements of the cytoskeleton, particularly actin microfilaments, are also involved in the initial stages of development and migration of OSNs. It has been established that OSNs originating in the basal regions of the epithelium after division of stem cells undergo a series of cytochemical rearrangements during their further migration to the upper layers of the OE. A wide layer of F-actin forms near the membrane at the iOSN surface along the dendrite. This densely packed network of actin microfilaments may have several functions. First, it ensures that secretory vesicles incorporate themselves only in the zone of the dendrite growth cone, leading to its extension; second, it stiffens the dendrite, enabling the cell to move between the bodies of other OE cells. Moreover, the cytoskeleton supports the actin-dependent transfer of the nucleus into the inner space of the dendrite for the reliable transport of the neuron between cells and towards the OE surface. When the cell acquires a “hot dog”-like shape with the penetration of nuclear material inside the actin casing of the dendrite, it likely becomes more streamlined in shape in order to migrate successfully within the epithelium. Examples of functional analogues of the identified actin-case structure include transmembrane actin-associated nuclear (TAN) lines and the perinuclear actin cap, which are directly involved in the transport and positioning of the nucleus in various types of cells *in vitro*^41,42^. This nuclear transfer may involve specialized adapter protein complexes that bind the nuclear envelope with actin microfilaments^43,44^. This process may also involve the ATP-dependent motor proteins myosins, which ensure the positioning of this organelle in the cytoplasm of somatic cells in many organisms^43,44^. These structural changes in young nerve cells might be versatile and might enable the transport and positioning of cells in other tissues as well as the OE. In the brain, where neurogenesis occurs^45,46^, newly formed neurons may undergo similar morphological reconstructions before their further migration.

Cytochemical studies show that actin microfilaments support not only the migration of cells and their positioning in the epithelium but also the formation of a specialized pore in the dense actin layer under the surface membrane of the dendritic terminal. *This suggests that*, *after contacting the environment*, *OSNs do not immediately participate in the complex processes of chemoreception of water-soluble odorants*. Naturally, a question arises: how can such a pore be formed at the top of the cell, in the compact layer of actin microfilaments? We could not detect the subsequent phases of dendrite integration into the surface of the olfactory epithelium. According to confocal microscopy data, the diameter of the apical section of a growing dendrite (where, as we have hypothesized, the dendrite cone growth is located) is approximately 0.90 ± 0.12 μm, while the pore diameter after it has been fixed in the olfactory epithelium is significantly smaller (0.37 ± 0.04 μm). Based on these observations, we can assume that, after the dendrite has made contact with the OE surface, actin polymerization processes are momentarily activated, and the surface area occupied by the cytoskeleton increases. This likely causes a partial contraction of the gap that does not contain actin microfilaments under the surface membrane of the olfactory knob, consequently causing a pore to form in this section.

Currently, it is difficult to provide a detailed description of the fine organization of this pore and to determine the variability of its structure. It is possible that, apart from actin, other cytoskeletal elements and functionally important components may be involved in maintaining the morphological integrity of the pore. However, the available data suggest that OSNs may interact with the environment through this pore and a respective fragment of the surface membrane.

These findings raise a number of questions: what is the functional purpose of this pore and the surface membrane fragment located above the pore? Can this membrane region concentrate ORs, ion channels, and enzymes that ensure the binding of odorant molecules and the subsequent transduction of extracellular signals? Based on the obtained data, we cannot make any assertions considering the time interval during which the pore can maintain a stable existence in the layer of actin microfilaments under the surface membrane of the top section of the OSN. Nevertheless, we could presume that, after it makes contact with the environment, an OSN can interact with a small fraction of odorants through a pore and thus guarantee that the signal will reach the cell. Therefore, if we consider that an iOSN can react to hydrophobic odorants during its development^29^, then after the first contact between the dendrite and the environment, the iOSN can interact with water-soluble molecules through a pore for the first time. Previously, it has been demonstrated for mammals that during OSN development, one *Olfr* allele is chosen for expression, and a coded receptor secures feedback that prevents the expression of other *Olfrs*^47–52^. Based on these data, it can be assumed that stabilization of chosen *Olfrs* occurs during pore formation in the OSN, which is an important insight for understanding the mechanism by which the OSN forms its receptor repertoire during its growth period.

Without further research, we cannot determine how micropores can participate in the structural-functional specialization of OSN. Nevertheless, in addition to the biochemical and genetic rearrangements typical of OSNs during differentiation, actin filaments play an important role in their development. In the early stages of OSN development, the actin cytoskeleton ensures a specific arrangement of the nuclear material inside the dendrite, allowing the migration of cells within the epithelium. When the dendrite makes contact with the epithelial surface, its terminal temporarily develops a dense layer of actin microfilaments with a pore through which the OSN can make its first contact with environmental odorants. The data obtained may be important for understanding the migration and differentiation of olfactory cells.

## Materials and Methods

### Animals

This study was performed on deep-water Baikal sculpins *Cottocomephorus inermis* (Jakowlew, 1890) (Cottidae) (Supplementary Fig. 1), n = 25. The sculpins were caught in southern Baikal at depths of 220–260 m with nets set under the ice in March–April 2008–2016. Their delivery to the surface required 30 min. After capture, the fishes were adapted in an aquarium with running Baikal water at a temperature of 4 °С. The data on their size, weight, sex, and age are shown in Table 1.

**Table 1:** Morphometric, weight, and sexual features of studied sculpin (Cottoidei) from Lake Baikal.

Species	n	L total, mm	Q, g	Sex	Age (years)
*C*. *inermis*	25	154–161	29.5–44.1	♀	4 + – 5+

All procedures performed on the fishes were carried out in compliance with the Helsinki Declaration of the World Medical Association (2000) and the EEC Directive 86/609 ЕЕС (1986) on the protection and welfare of experimental animals. All experimental procedures were approved by the Bioethics Committee with the Scientific Council of the Biology Department, Irkutsk State University, on November 30, 2007 (Permit No. 3).

### Transmission electron microscopy

Olfactory rosettes were fixed in 2.5% glutaraldehyde (Sigma-Aldrich, United States) solution in 0.1 M phosphate buffer, pH 7.3 at 4 °C for 2 h, postfixed in 2% OsO4 (Merck, Germany) in the same buffer for 12 h, dehydrated in an ascending alcohol gradient with acetone, and embedded in Araldite 502 resin with a DMP-30 accelerator (Araldite 502 Kit, SPI Supplies, United States). Ultrathin sections (70*–*80 nm) made with an Ultracut R microtome (Leica, Germany) were examined under a Leo 906E transmission electron microscope at an accelerating voltage of 80 kV. Microscopic images made with a MegaView II digital camera were processed using the MegaVision programme package (Soft Imaging System GmbH, Germany).

### Laser confocal microscopy

The olfactory rosettes of *C*. *inermis* are small in size (2–3 mm), preventing their division into separate petals. Therefore, neurogenesis and the cytochemical features of the differentiation of OSNs were investigated by confocal microscopy on integral olfactory organs.

Proliferating cells in the olfactory epithelium were detected with an FITC BrdU Flow Kit (Becton, Dickinson and Company BD Biosciences, USA, Cat. No. 559619). This method is based on the incorporation of bromodeoxyuridine (BrdU) into DNA during the S-phase of the cell cycle^53,54^. The BrdU incorporated into the newly synthesized DNA is stained with FITC-conjugated anti-BrdU antibody. Total cell DNA is stained with 7-aminoactinomycin^55^. Proliferating cells in olfactory rosettes were labelled *in vivo*. Fishes were given an intraperitoneal injection of BrdU in Dulbecco’s phosphate buffered saline (0.5 mg per 10 g body mass). The olfactory rosettes were retrieved for the detection of BrdU-positive cells 12 hours after the BrdU injection. The fixation, permeabilisation, and staining of olfactory epithelial nuclei for the detection of BrdU labelling were performed according to the FITC BrdU Flow Kit Guidelines. The nuclei was analysed using an LSM 710 laser confocal microscope (Carl Zeiss); lens: Plan-Apochromat 20 × /0.8 M27; lasers: 561 nm: 5.0% and 488 nm: 5.0%.

Functionally active mitochondria in olfactory epithelial cells were stained with MitoTracker® Orange CMTMRos (Thermo Fisher Scientific Inc., USA, Cat. No. M7510) by 25-min incubation in medium 199 with Hank’s salts (Kompaniya PanEko, Russia, Cat. No. S230p); the medium contained 100–500 nM dye at 37 °С. The olfactory rosettes were fixed for 15 min in a 2% paraformaldehyde (Sigma-Aldrich Co. LLC, USA, Cat. No. 158127) solution in 0.1 M phosphate buffer (pH 7.4) and permeabilised in 0.25% Triton™ X-100 (Sigma-Aldrich Co. LLC, USA, Cat. No. T8787) for 25 min. Actin microfilaments were stained with FITC-Phalloidin (Sigma-Aldrich Co. LLC, USA, Cat. No. P5282) for 40 min^56^. Cell nuclei were stained with DAPI (Sigma-Aldrich Co. LLC, USA, Cat. No. D9542) for 15 min. After each step, the samples were washed three times in Hank’s solution without phenol red (PanEko, Russia, Cat. No Р020p). The stained samples were mounted on glass slides in ProLong® Gold Antifade Mountant (Thermo Fisher Scientific Inc., USA, Cat. No. P36930) and covered with a coverslip. The slides were analysed using an LSM 710 laser confocal microscope (Carl Zeiss); lens: Plan-Apochromat 63×/1.40 Oil DIC M27; lasers: track 1, 405 nm: 3.0%; track 2, 488 nm: 3.0%; track 3, 561 nm: 3.0%.

To assess the proliferative activity of OE cells, we obtained 15–20 Z-stacks from each olfactory rosette. The number of BrdU incorporating cells in the tissue was estimated using the Zen 2010 (Carl Zeiss) and Imaris® Bitplane 7.2.3 software package (Bitplane AG, Switzerland). The Z-stacks of the images of olfactory folds were divided into separate, smaller (1 × 10^5^ μm^3^) fragments. To estimate the volume of tissue in a Z-stack, we estimated the volume occupied by the 7-AAD signal staining the entire nuclear tissue. In each tissue volume of Z-stacks, we counted the number of nuclei that incorporated BrdU (based on the binding of FITC-conjugated antibodies to BrdU). Based on the obtained data, we calculated for each Z-stack the number of nuclei in 1 × 10^6^ μm^3^ of tissue. We processed all the information obtained for each of the fragments using parametric statistics, and we calculated the mean and standard deviation using the Statistica 10 software package.

To analyse actin microfilaments, mitochondria, and nuclear material in OSNs, we obtained 15–20 Z-stacks from each olfactory rosette. The average thickness of the Z-stacks was 20–30 μm. For this purpose, we examined the olfactory rosettes layer by layer by 2D cuts, bulk Z-stacks, or by making orthogonal projections of selected areas.

Cytochemical data on the OSN cytoskeleton that has been detected by confocal microscopy was compared with the morphological features of OSNs, studied earlier with electron microscopy in *C*. *inermis*^23^. The olfactory epithelium has ciliated supporting cells (cSC) and secretory supporting cells (sSC). Similar to other fish species, both SC types permeate the entire epithelium and emerge at the epithelial surface. The diameter of cell body, including the apical section, is 4–5 µm. The top of a cSC includes 20–30 cilia, which contain large quantities of mitochondria at their bases. The sSC cytoplasmic matrix is usually well-visualized and can contain granules of various densities. Ciliated and microvillar OSNs interspersed with SCs are detected in the OE. In contrast to SCs, OSNs have a spindle-like form. The dendrite diameter varies from 3.7 μm near the nucleated cell compartment to 2 μm when contacting the OE surface. Ciliated OSNs at the dendritic knob contain 4–5 cilia; 8-10 short microvilli usually branch from the top of microvillar OSNs.

## Electronic supplementary material

Video 1

Video 2

Video 3

Video 4

Supplementary Figure 1
