# Supplementary material for: Rearrangement of Actin Microfilaments in the Development of Olfactory Receptor Cells in Fish
Source: Sci Rep. 2018 Feb 27;8:3692. doi: 10.1038/s41598-018-22049-7 (PMC5829147; doi:10.1038/s41598-018-22049-7)
Supplement: Supplementary file 5 — Supplementary Figure 1 [file 41598_2018_22049_MOESM5_ESM.doc]

**REARRANGEMENT OF ACTIN MICROFILAMENTS IN THE DEVELOPMENT**

**OF OLFACTORY RECEPTOR CELLS IN FISH**

Igor V. Klimenkov^1,2*^, Nikolay P. Sudakov^1,2,3^, Mikhail V. Pastukhov^4^, Mikhail M. Svinov^5^, and Nikolay S. Kositsyn^5^

^1^ Limnological Institute, Siberian Branch, Russian Academy of Sciences, 3 Ulan-Batorskaya St., Irkutsk, 664033 Russia

^2^ Irkutsk State University, 1 Karl Marx St., Irkutsk, 664003 Russia

^3^ Irkutsk Scientific Center of Surgery and Traumatology, 1 Bortsov Revolyutsii St., Irkutsk, 664003 Russia

^4^ Vinogradov Institute of Geochemistry, Siberian Branch, Russian Academy of Sciences, 1a Favorsky St., Irkutsk, 664033 Russia

^5^ Institute of Higher Nervous Activity and Neurophysiology, Russian Academy of Sciences, 5a Butlerova St., Moscow, 117485 Russia


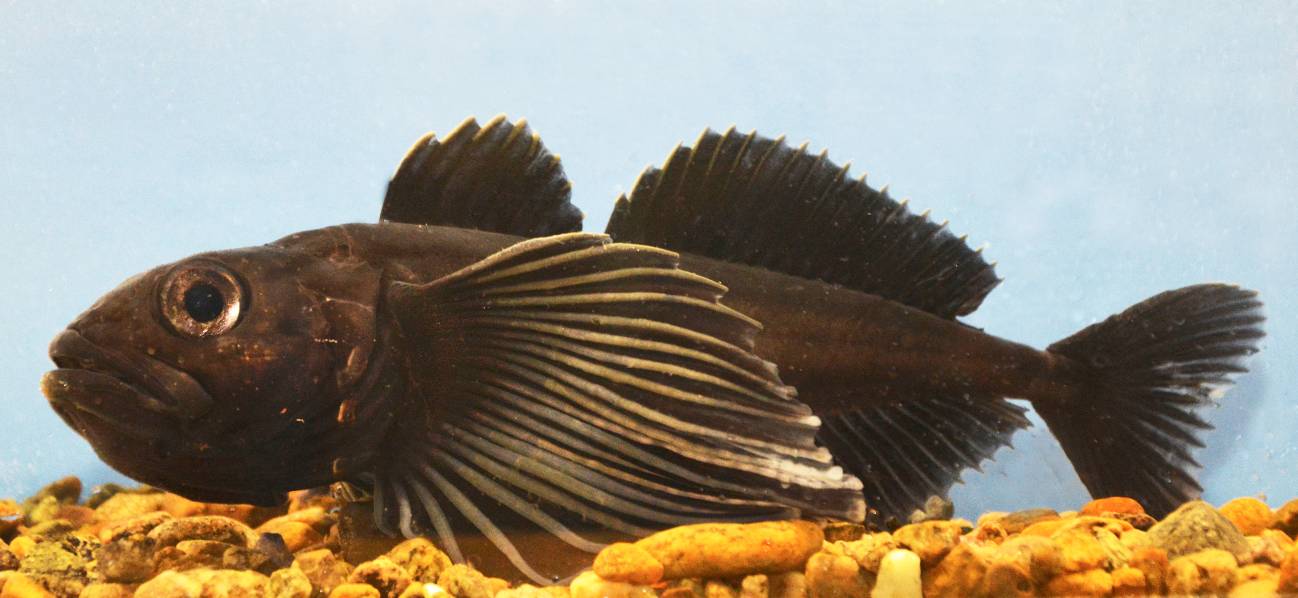


**Supplementary Figure 1.** *Cottocomephorus inermis* (Jakowlew, 1890) (Cottidae)*,* endemic to Lake Baikal.
